# The preoperative sensitive-modified Glasgow prognostic score is superior to the modified Glasgow prognostic score in predicting long-term survival for esophageal squamous cell carcinoma

**DOI:** 10.18632/oncotarget.11268

**Published:** 2016-08-12

**Authors:** Rui Tian, Fei Zhang, Peng Sun, Jing Wu, Hong Yan, Ai-Ran Wu, Min Zhang, Yu-Lu Jiang, Yan-Hong Lu, Qiu-Yan Xu, Xiao-Hong Zhan, Rong-Xin Zhang, Li-Ting Qian, Jie He

**Affiliations:** ^1^ Department of Pathology, Anhui Cancer Hospital & Anhui Provincial Hospital Affiliated Anhui Medical University, Hefei, Anhui, People's Republic of China; ^2^ Collaborative Innovation Center for Cancer Medicine, Guangzhou, Guangdong, People's Republic of China; ^3^ State Key Laboratory of Oncology in South China, Guangzhou, Guangdong, People's Republic of China; ^4^ Department of Medical Oncology, Sun Yat-sen University Cancer Center, Guangzhou, Guangdong, People's Republic of China; ^5^ Department of Thoracic Surgery, Anhui Cancer Hospital & Anhui Provincial Hospital Affiliated Anhui Medical University, Hefei, Anhui, People's Republic of China; ^6^ Department of Radiology, Anhui Provincial Hospital & Anhui Provincial Hospital Affiliated Anhui Medical University, Hefei, Anhui, People's Republic of China

**Keywords:** esophageal squamous cell carcinoma, modified Glasgow prognostic score, superiority, survival

## Abstract

The present study was designed to investigate the prognostic significance of the preoperative sensitive-modified Glasgow prognostic score (S-mGPS) and its superiority in esophageal squamous cell carcinoma (ESCC). Clinicopathologic characteristics, preoperative albumin and C-reactive protein (CRP) levels were retrospectively collected in 442 patients who underwent transthoracic esophagectomy. The S-mGPS was calculated before surgery based on optimal cutoff values of 45.6 g/L for albumin and 10.0 mg/L for CRP. 360, 74 and 8 cases were assigned an mGPS of 0, 1 and 2, respectively. In contrast, the S-mGPS was 0 in 114, 1 in 258 and 2 in 70 patients. Of the 360 patients with an mGPS of 0, 246 migrated to the S-mGPS-1 group. Both mGPS and S-mGPS were significantly correlated with tumor length, depth of invasion, pathological tumor-node-metastasis (pTNM) stage and adjuvant treatment. In addition, they were significantly associated with disease free survival (DFS) and overall survival (OS) in univariate analysis. Furthermore, multivariate Cox regression analysis identified S-mGPS as an independent prognostic indicator for both DFS [hazard ratio (HR), 1.577; 95% confidence interval (CI), 1.149-2.163; *P* = 0.005] and OS (HR, 1.762; 95% CI, 1.250-2.484; *P* = 0.001), but not mGPS (HR, 0.957; 95% CI, 0.692-1.323; *P* = 0.790 for DFS and HR, 1.089; 95% CI, 0.781-1.517; *P* = 0.615 for OS, respectively). Moreover, subgroup analysis revealed that the prognostic impact of the S-mGPS was especially striking in pTNM stage II patients. The preoperative S-mGPS is superior to the mGPS as a prognostic predictor in patients with resectable ESCC.

## INTRODUCTION

Esophageal cancer is the sixth most common malignancy and fourth highest cause of cancer-related death in China [1]. It has two main pathological subtypes: esophageal squamous cell carcinoma (ESCC) and esophageal adenocarcinoma (EAC). ESCC is the predominant subtype in certain regions of China [2–5]. Esophagectomy with lymph node dissection is a potentially curative treatment modality, however, the prognosis remains poor, with a reported 5-year overall survival (OS) rate of less than 40% [3–5].

Modified Glasgow prognostic score (mGPS), which is defined based on the serum concentrations of C-reactive protein (CRP) and albumin [6], has been identified as a prognostic predictor in various malignancies, including ESCC [7–10]. Zhang et al. suggested that mGPS was an independent prognostic indicator for overall survival (OS) and progression free survival (PFS) in inoperable thoracic ESCC patients undergoing chemoradiotherapy [7]. However, several recent studies failed to identify its independent prognostic significance in ESCC patients who underwent esophagectomy [11–12]. Just as they indicated, as the majority of the included patients were classified in the group of a score of 0, mGPS could not distinguish the survival difference of most of the cases.

Previous studies have indicated that albumin levels were not always decreased in operable esophageal cancer patients, making nutritional deficiency insufficient for risk stratification [13–15]. In addition, the preoperative albumin-globulin score (AGS) determined with a cutoff value of 45.6 g/L for albumin has been identified as an independent predictor for OS in resectable ESCC [11]. Thus, we hypothesized that a more sensitive mGPS (S-mGPS) by defining an albumin cutoff value of 45.6 g/L in addition to the mGPS might show more superiority in predicting the long-term survival.

Therefore, the purpose of the present study was to calculate and compare the prognostic significance of the preoperative mGPS and S-mGPS in patients with ESCC who underwent esophagectomy with lymphadenectomy.

## PATIENTS AND METHODS

### Patients

A total of 442 patients with histopathologically diagnosed ESCC underwent radical transthoracic esophagectomy at the Department of Thoracic Surgery, Anhui Cancer Hospital and Anhui Provincial Hospital between January 2005 and December 2010 were included in this study. Patients who received preoperative chemotherapy or chemoradiotherapy were excluded. And this study was approved by the independent ethics committees at our institution and was performed in accordance with the ethical standards of the World Medical Association Declaration of Helsinki.

### Treatment and follow up

The surgical procedures included both left and right transthoracic esophagectomy with curative intent, and at least a two-field regional lymphadenectomy, including standard, extended, or total dissection of the cervical, thoracic and abdominal lymph nodes, was performed. And the median number of dissected lymph nodes was 19 (range, 12-89). Adjuvant treatment was planned according to the tumor stage, doctor's selection and patient's desire. All patients were regularly followed with physical examination, upper gastrointestinal endoscopy, tumor marker and computed tomography every 3 months for the first 2 years, every 6 months in the third year and yearly thereafter for more than 5 years.

### Clinicopathologic and laboratory parameters

Patients’ baseline clinicopathologic and laboratory variables including preoperative serum albumin and CRP levels were retrospectively reviewed and collected from the electronic medical records. The tumor stage was determined according to the pathological tumor-node-metastasis (pTNM) classification of the International Union Against Cancer (UICC), seventh edition. The tumor length was defined as the long diameter of the general post-operative pathological specimens. The degree of differentiation was categorized into poorly/not differentiated, moderately differentiated and well differentiated. And the tumor locations were classified into upper esophagus, middle esophagus and lower esophagus. The preoperative serum concentrations of albumin and CRP were measured by an automatic biochemical analyzer (Hitachi 7600, Japan) within 3 days prior to surgery.

### Definition of the modified Glasgow Prognostic Score (mGPS) and sensitive mGPS (S-mGPS)

The mGPS was determined as previously described: patients with an elevated CRP (> 10.0 mg/L) level and decreased albumin (< 35.0 g/L) were assigned a score of 2, those with both elevated CRP and albumin (> 35.0 g/L) levels were allocated a score of 1 and those with a normal CRP level (< 10.0 mg/L) were given a score of 0.

As for S-mGPS in the present study, the optimal cut-off value for serum albumin was set at 45.6 g/L based on the receiver operating characteristic (ROC) curve analysis, which was in accordance with the previous report by Zhang et al. [11]. Patients with elevated CRP (> 10.0 mg/L) and decreased albumin (< 45.6 g/L) levels were allocated a score of 2, those with only one of these two abnormalities were assigned a score of 1, and those with neither of the two abnormalities were classified as having a score of 0.

### Statistical analysis

Chi-square test, Mann-Whitney U test or Kruskal-Wallis test was used to examine the differences of baseline and clinicopathologic characteristics between groups. Disease free survival (DFS) was defined as the date of surgery to local recurrence/distant metastasis or to the last date of follow-up, OS was the time interval from the date of surgery to death from ESCC or to the most recent follow-up. Survival curves were estimated with the Kaplan-Meier method, and differences were compared using the log-rank test. Univariate and multivariate analysis were performed using Cox proportional hazards regression models and hazard ratios (HRs) for variables respecting to DFS and OS were calculated. All statistical analyses were performed with SPSS 16.0 (SPSS Inc., Chicago, IL, USA). And a two-sided *P* value of < 0.05 was considered statistically significant.

## RESULTS

### Baseline clinicopathologic characteristics

Of the patients, 331 (74.9%) were males and 111 (25.1%) were females, with a median age of 60.0 years (range, 20.0-88.0 years). The primary tumors were located at the middle esophagus in 277 (62.7%) cases. The histopathological type was moderately differentiated in 227 (51.5%) cases. And the tumor was classified as stage I in 40 (9.0%) patients, stage II in 209 (47.3%) patients and stage III in 193 (43.7%) patients, respectively (Table 1).

**Table 1:** Correlation between preoperative mGPS, S-mGPS and clinicopathological characteristics in 442 ESCC patients

Clinicopathologic characteristics	Number of patients (N, %)	mGPS (N, %)			*P* value	S-mGPS (N, %)			*P* value
		0	1	2		0	1	2	
Age (years)					0.757				0.001*
< 60	262 (59.3)	216 (60.0)	42 (56.8)	4 (50.0)		80 (70.2)	144 (55.8)	38 (54.3)	
≥ 60	180 (49.7)	144 (40.0)	32 (43.2)	4 (50.0)		34 (29.8)	114 (44.2)	32 (45.7)	
Gender					0.707				0.371
Male	331 (74.9)	271 (75.3)	55 (74.3)	5 (62.5)		90 (78.9)	187 (72.5)	54 (77.1)	
Female	111 (25.1)	89 (24.7)	19 (25.7)	3 (37.5)		24 (21.1)	71 (27.5)	16 (22.9)	
Tumor lacation					0.093				0.423
Upper	39 (8.8)	30 (8.3)	9 (12.2)	0 (0.0)		7 (6.1)	23 (8.9)	9 (12.9)	
Middle	277 (62.7)	233 (64.8)	37 (50.0)	7 (87.5)		76 (66.7)	163 (63.2)	38 (54.3)	
Lower	126 (28.5)	97 (26.9)	28 (37.8)	1 (12.5)		31 (27.2)	72 (27.9)	23 (32.8)	
Tumor length (cm)					0.001*				< 0.001*
< 5	236 (53.4)	207 (57.5)	26 (35.1)	3 (37.5)		74 (64.9)	140 (54.3)	22 (31.4)	
≥ 5	206 (46.6)	153 (42.5)	48 (64.9)	5 (62.5)		40 (35.1)	118 (45.7)	48 (68.6)	
Differentiation					0.380				0.836
Well	115 (26.0)	92 (25.6)	21 (28.4)	2 (25.0)		30 (26.3)	66 (25.6)	19 (27.1)	
Moderate	227 (51.4)	189 (52.5)	36 (48.6)	2 (25.0)		56 (49.1)	138 (53.5)	33 (47.1)	
Poor/Undifferentiated	100 (22.6)	79 (21.9)	17 (23.0)	4 (50.0)		28 (24.6)	54 (20.9)	18 (25.8)	
pT stage					< 0.001*				< 0.001*
T1	42 (9.5)	40 (11.1)	2 (2.7)	0 (0.0)		18 (15.8)	22 (8.5)	2 (2.9)	
T2	70 (15.8)	65 (18.1)	4 (5.4)	1 (12.5)		24 (21.1)	42 (16.3)	4 (5.8)	
T3	296 (67.0)	235 (65.2)	56 (75.7)	5 (62.5)		67 (58.8)	178 (69.0)	51 (72.8)	
T4	34 (7.7)	20 (5.6)	12 (16.2)	2 (25.0)		5 (4.3)	16 (6.2)	13 (18.5)	
pN stage					0.103				0.206
N0	232 (52.5)	200 (55.6)	28 (37.8)	4 (50.0)		67 (58.8)	137 (53.1)	28 (40.0)	
N1	117 (26.5)	92 (25.6)	22 (29.7)	3 (37.5)		26 (22.7)	67 (26.0)	24 (34.3)	
N2	73 (16.5)	54 (15.0)	18 (24.4)	1 (12.5)		19 (16.7)	40 (15.5)	14 (20.0)	
N3	20 (4.5)	14 (3.8)	6 (8.1)	0 (0.0)		2 (1.8)	14 (5.4)	4 (5.7)	
pTNM stage					< 0.001*				< 0.001*
I	40 (9.0)	38 (10.6)	2 (2.7)	0 (0.0)		18 (15.8)	20 (7.8)	2 (2.9)	
II	209 (47.3)	182 (50.6)	23 (31.1)	4 (50.0)		55 (48.2)	131 (50.7)	23 (32.9)	
III	193 (43.7)	140 (38.8)	49 (66.2)	4 (50.0)		41 (36.0)	107 (41.5)	45 (64.2)	
Smoking					0.337				0.825
Never	166 (37.6)	134 (37.2)	27 (36.5)	5 (62.5)		41 (36.0)	100 (38.8)	25 (35.7)	
Ever	276 (62.4)	226 (62.8)	47 (63.5)	3 (37.5)		73 (64.0)	158 (61.2)	45 (64.3)	
Alcohol consumption					0.280				0.025*
Never	291 (65.8)	239 (66.4)	45 (60.8)	7 (87.5)		64 (56.1)	182 (70.5)	45 (64.3)	
Ever	151 (34.2)	121 (33.6)	29 (39.2)	1 (12.5)		50 (43.9)	76 (29.5)	25 (35.7)	
Adjuvant treatment					0.001*				0.008*
Yes	76 (17.2)	50 (13.9)	23 (31.1)	3 (37.5)		16 (14.0)	39 (15.1)	21 (30.0)	
No	366 (82.8)	310 (86.1)	51 (68.9)	5 (62.5)		98 (86.0)	219 (84.9)	49 (70.0)	

mGPS, modified Glasgow prognostic score; S-mGPS, sensitive-modified Glasgow prognostic score; ESCC, esophageal squamous cell carcinoma; TNM, tumor-node-metastasis.

* *P* < 0.05.

### Correlation between preoperative mGPS, S-mGPS and clinicopathologic parameters

Of the included 442 patients, 360 had a preoperative mGPS of 0, 74 had an mGPS of 1 and 8 had an mGPS of 2. The results demonstrated that the mGPS was significantly correlated with tumor length, pT stage, pTNM stage and adjuvant treatment (Table 1). In contrast, 114 (25.8%), 258 (58.4%) and 70 (15.8%) patients were classified as having an S-mGPS of 0, 1 and 2, respectively (Table 1). Moreover, of the 360 patients with an mGPS of 0, 246 migrated to the S-mGPS-1 group, whereas none exhibited an S-mGPS of 2 (Table 2). The S-mGPS was confirmed to be significantly associated with age, tumor length, pT stage, pTNM stage, alcohol consumption and adjuvant treatment (Table 1).

**Table 2:** Univariate and multivariate analysis of DFS in 442 ESCC patients

Variables	Univariate			Multivariate		
	HR	95% CI	*P*	HR	95% CI	*P*
Age (years)						
≥ 60	1		0.996			NI
< 60	0.996	0.779-1.273				
Gender						
Male	1		0.040*	1		0.920
Female	0.736	0.550-0.986		0.979	0.642-1.491	
Tumor location						
Lower	1		0.781			NI
Middle	0.984	0.747-1.297				
Upper	1.144	0.726-1.802				
Tumor length (cm)						
< 5	1		0.001*	1		0.152
≥ 5	1.501	1.177-1.913		1.204	0.934-1.553	
Differentiation						
Well/Moderate	1		0.113			NI
Poor/Undifferentiated	1.253	0.948-1.657				
Depth of invasion						
T1/T2	1		< 0.001*			NI
T3/T4	2.06	1.501-2.827				
Lymph node involvement						
Negative	1		< 0.001*			NI
Positive	2.753	2.141-3.539				
TNM stage						
I/II	1		< 0.001*	1		< 0.001*
III	2.371	1.854-3.033		2.081	1.596-2.713	
Smoking						
Never	1		0.035*	1		0.764
Ever	1.318	1.019-1.703		1.06	0.724-1.552	
Alcohol consumption						
Never	1		0.010*	1		0.123
Ever	1.391	1.083-1.786		1.256	0.940-1.679	
Adjuvant treatment						
Yes	1		0.006*	1		0.543
No	1.529	1.133-2.065		1.104	0.803-1.518	
mGPS						
0	1		0.025*	1		0.790
1/2	1.405	1.044-1.893		0.957	0.692-1.323	
S-mGPS						
0	1		0.001*	1		0.005*
1/2	1.702	1.259-2.302		1.577	1.149-2.163	

DFS, overall survival.

* *P* < 0.05.

### Prognostic value of the preoperative mGPS and S-mGPS in predicting long-term survival for ESCC

Two hundred and thirty-five patients died during the follow-up period, with an estimated median DFS and OS of 35.6 months (95%CI, 24.3-46.9 months) and 57.4 months (95%CI, 37.8-77.0 months), respectively.

**Figure 1:**
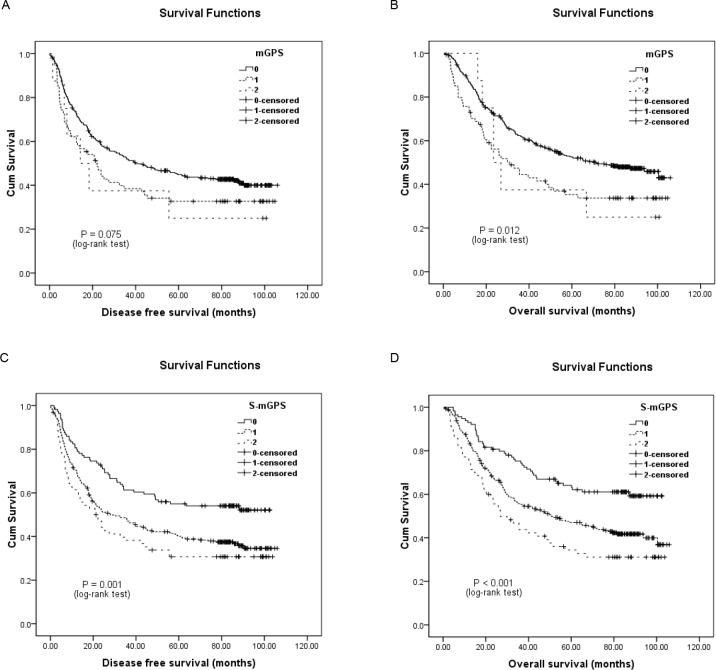
Kaplan-Meier survival curves of 442 esophageal squamous cell carcinoma patients **A.**, disease-free survival (DFS) and **B.**, overall survival (OS) stratified by their preoperative modified Glasgow prognostic scores (mGPS); C, DFS and D, OS stratified by their preoperative sensitive-modified Glasgow prognostic scores (S-mGPS).

Univariate survival analysis for DFS demonstrated that both high preoperative mGPS (HR, 1.405; 95%CI, 1.044-1.893; *P* = 0.025; Figure 1A) and S-mGPS (HR, 1.702; 95%CI, 1.259-2.302; *P* = 0.001; Figure 1C) were significantly associated with unfavorable DFS. Gender (Male/Female), tumor length (< 5/≥5 cm), depth of invasion (T1-2/T3-4), lymph node involvement (Negative/Positive), pTNM stage (I-II/III), smoking (Never/Ever), alcohol consumption (Never/Ever), adjuvant treatment (Yes/No) were other significant prognostic parameters (*P* < 0.05). After adjusting for other confounding variables, the multivariate Cox proportional hazards model revealed that S-mGPS (HR, 1.577; 95% CI, 1.149-2.163; *P* < 0.001) and pTNM stage (HR, 2.081; 95% CI, 1.596-2.713; *P* < 0.001) were two independent predictors for DFS. Unfortunately, mGPS failed to be an independent prognostic indicator for DFS (HR, 0.957; 95% CI, 0.692-1.323; *P* = 0.790) (Table 3).

**Table 3:** Univariate and multivariate analysis of OS in 442 ESCC patients

Variables	Univariate			Multivariate		
	HR	95% CI	*P*	HR	95% CI	*P*
Age (years)						
≥ 60	1		0.370			NI
< 60	1.125	0.869-1.456				
Gender						
Male	1		0.068			NI
Female	0.752	0.553-1.022				
Tumor location						
Lower	1		0.794			NI
Middle	1.091	0.810-1.471				
Upper	1.157	0.704-1.901				
Tumor length (cm)						
< 5	1		0.004*	1		0.431
≥ 5	1.450	1.122-1.873		1.113	0.852-1.454	
Differentiation						
Well/Moderate	1		0.122			NI
Poor/Undifferentiated	1.261	0.940-1.691				
Depth of invasion						
T1/T2	1		< 0.001*			NI
T3/T4	2.459	1.723-3.510				
Lymph node involvement						
Negative	1		< 0.001*			NI
Positive	2.727	2.090-3.558				
TNM stage						
I/II	1		< 0.001*	1		< 0.001*
III	2.494	1.921-3.237		2.189	1.657-2.893	
Smoking						
Never	1		0.056			NI
Ever	1.302	0.993-1.708				
Alcohol consumption						
Never	1		0.002*	1		0.005*
Ever	1.508	1.160-1.961		1.465	1.121-1.915	
Adjuvant treatment						
Yes	1		0.007*	1		0.699
No	1.541	1.124-2.112		1.068	0.765-1.491	
mGPS						
0	1		0.003*	1		0.615
1/2	1.583	1.165-2.151		1.089	0.781-1.517	
S-mGPS						
0	1		< 0.001*	1		0.001*
1/2	1.875	1.351-2.603		1.762	1.250-2.484	

OS, overall survival.

* *P* < 0.05.

As for OS, patients with high preoperative mGPS (HR, 1.583; 95% CI, 1.165-2.151; *P* = 0.003; Figure 1B) and S-mGPS (HR, 1.875; 95% CI, 1.351-2.603; *P* < 0.001; Figure 1D) tended to have impaired OS by univariate analysis. Besides, other variables including tumor length, depth of invasion, lymph node involvement, pTNM stage, alcohol consumption and adjuvant treatment could also predict OS. Further multivariate analysis identified preoperative S-mGPS (HR, 1.762; 95% CI, 1.250-2.484; *P* = 0.001) and pTNM stage (HR, 2.189; 95% CI, 1.657-2.893; *P* < 0.001) as independent prognostic factors for OS, but not mGPS (HR, 1.089; 95% CI, 0.781-1.5173; *P* = 0.615) (Table 4).

**Figure 2:**
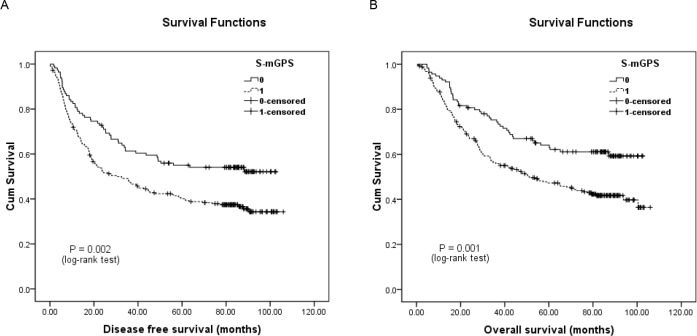
Kaplan-Meier survival curves of A., DFS and B., OS in the mGPS-0 group (*N* = 360) patients classified by their preoperative S-mGPS levels

**Figure 3:**
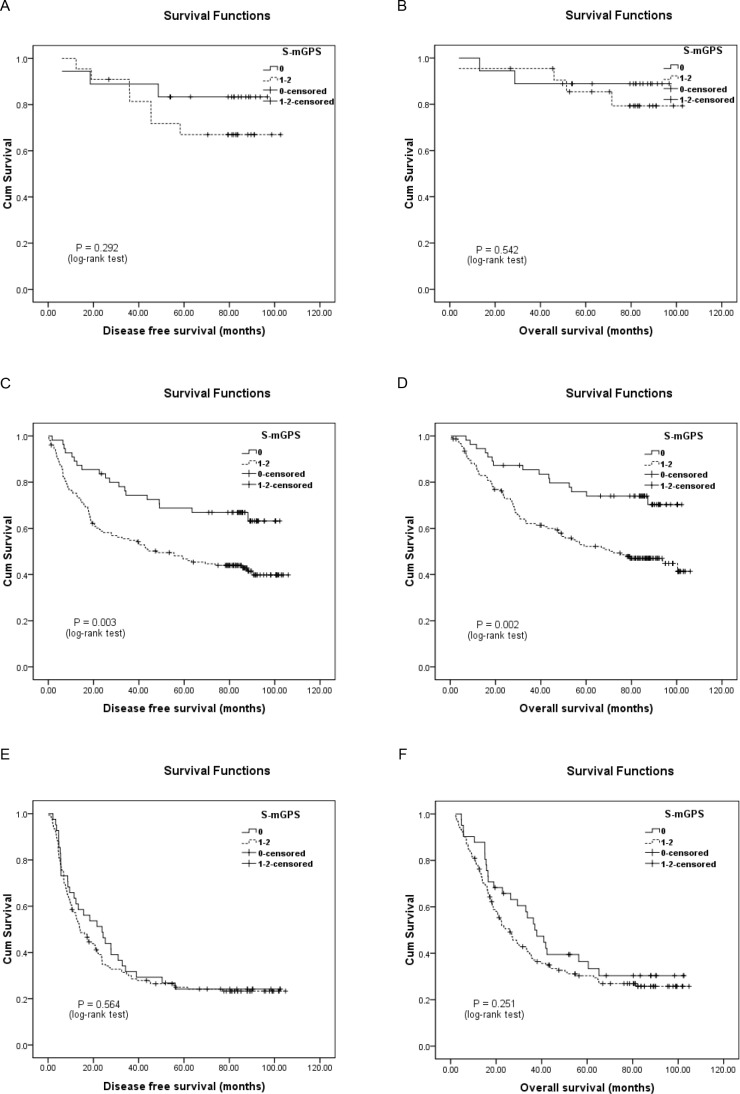
Kaplan-Meier survival curves of A., DFS and B., OS in pathological tumor-node-metastasis (pTNM) stage I patients (*N* = 40); C., DFS and D., OS in pTNM stage II patients (*N* = 209); E., DFS and F, OS in pTNM stage III patients (*N* = 193) stratified by their preoperative S-mGPS levels

In addition, the 246 patients who migrated to the S-mGPS-1 group demonstrated significantly more poorer DFS and OS than those who remained in the S-mGPS-0 group (Figure 2). Moreover, subgroup analysis based on different pTNM stages indicated that high preoperative S-mGPS was significantly correlated with unfavorable DFS and OS in pTNM stage II patients (Figure 3C-3D; *P* < 0.05), but not in pTNM stage I or III patients (Figure 3A-3B, 3E-3F; *P* > 0.05).

## DISCUSSION

mGPS, a cumulative prognostic score based on cutoff values of 35.0 g/L for serum albumin and 10.0 mg/L for CRP, was first advocated by McMillan with the aim to more accurately predict the prognosis of various malignancies [6]. However, the weak point of this scoring system was that patients with abnormal mGPS levels were the minority, resulting in its ability to identify a special small cohort of patients with an impaired prognosis [11–12, 16]. Actually, of the 442 patients in the present study, the vast majority of the cases were classified to the mGPS-0 group, whereas only 82 (18.6%) were assigned an mGPS of 1 or 2, which was in accordance with prior studies [11–12].

To address this weak point, this study adopted the more sensitive cutoff value of 45.6 g/L for albumin, which was determined based on the ROC curves and reported by Zhang et al. as an alternative [11]. The multivariate Cox regression analysis revealed that the preoperative S-mGPS could serve as an independent prognostic indicator in comparison with the mGPS. Besides, of the 360 cases with an mGPS of 0, 246 migrated to the S-mGPS-1 group and they demonstrated significantly unfavorable prognosis when compared to those with an S-mGPS of 0, suggesting that the S-mGPS could predict the prognosis more sensitively than the mGPS. Additionally, the S-mGPS could also be utilized to identify patients who were with significantly poorer prognosis in the mGPS-0 group. Furthermore, subgroup analysis suggested that the preoperative S-mGPS demonstrated significant prognostic difference in pTNM stage II patients, indicating that further intense adjuvant treatment should be considered in certain patients. However, no survival difference was identified in pTNM stage I or III patients, although standard therapeutic strategies for these patients remain critical and controversial. It might be the limitation of the preoperative S-mGPS as a prognostic factor in patients with resectable ESCC.

Previous studies have demonstrated that decreased albumin levels could not be commonly observed in early or locally advanced esophageal caner patients, leaving it insufficient for risk classification [13–15]. Recently, two studies observed better nutrition status in operable esophageal cancer patients than in those with metastatic disease [11, 15]. Matsuda et al. defined the serum albumin cut-off value as 38.0 g/L according to the lower quartile range, and showed that the prognostic indicator based on preoperative plasma fibrinogen and serum albumin levels (FA score) was remarkably predictive of postoperative survival in esophageal cancer patients [15]. Meanwhile, Zhang and his colleagues established the preoperative albumin-globulin score (AGS) based on a cutoff value of 45.6 g/L for albumin, and identified its independent prognostic significance for OS in resectable ESCC [11]. Thus, the cut-off value of 45.6 g/L was used in the present study, and the multivariate survival analysis determined pTNM stage and the preoperative S-mGPS as independent prognostic indicators for DFS and OS.

Although this study was designed retrospectively and performed in a single-center, the present data strongly suggested that the preoperative S-mGPS could serve as an independent prognostic indicator and was superior to the mGPS in predicting long-term survival for ESCC patients who underwent transthoracic esophagectomy. However, it is still necessary to perform further prospective multicenter cohort studies to validate these findings.

## References

[1] Chen W, Zheng R, Baade PD, Zhang S, Zeng H, Bray F, Jemal A, Yu XQ, He J (2016). Cancer statistics in China, 2015. CA Cancer J Clin.

[2] Arnold M, Soerjomataram I, Ferlay J, Forman D (2015). Global incidence of oesophageal cancer by histological subtype in 2012. Gut.

[3] Lin Y, Totsuka Y, He Y, Kikuchi S, Qiao Y, Ueda J, Wei W, Inoue M, Tanaka H (2013). Epidemiology of esophageal cancer in Japan and China. J Epidemiol.

[4] Sun P, Zhang F, Chen C, An X, Li YH, Wang FH, Zhu ZH (2013). Comparison of the prognostic values of various nutritional parameters in patients with esophageal squamous cell carcinoma from Southern China. J Thorac Dis.

[5] Sun P, Zhang F, Chen C, Bi X, Yang H, An X, Wang F, Jiang W (2016). The ratio of hemoglobin to red cell distribution width as a novel prognostic parameter in esophageal squamous cell carcinoma: a retrospective study from southern china. Oncotarget.

[6] McMillan DC, Crozier JE, Canna K, Angerson WJ, McArdle CS (2007). Evaluation of an inflammation-based prognostic score (GPS) in patients undergoing resection for colon and rectal cancer. Int J Colorectal Dis.

[7] Zhang P, Xi M, Li QQ, He LR, Liu SL, Zhao L, Shen JX, Liu MZ (2014). The modified Glasgow Prognostic Score is an independent prognostic factor in patients with inoperable thoracic esophageal squamous cell carcinoma undergoing chemoradiotherapy. J Cancer.

[8] Proctor MJ, Morrison DS, Talwar D, Balmer SM, O'Reilly DS, Foulis AK, Horgan PG, McMillan DC (2011). An inflammation-based prognostic score (mGPS) predicts cancer survival independent of tumour site: a Glasgow Inflammation Outcome Study. Br J Cancer.

[9] Nakagawa K, Tanaka K, Nojiri K, Kumamoto T, Takeda K, Ueda M, Endo I (2014). The modified Glasgow prognostic score as a predictor of survival after hepatectomy for colorectal liver metastasis. Ann Surg Oncol.

[10] Fan H, Shao ZY, Xiao YY, Xie ZH, Chen W, Xie H, Qin GY, Zhao NQ (2016). Comparison of the Glasgow Prognostic Score (GPS) and the modified Glasgow Prognostic Score (mGPS) in evaluating the prognosis of patients with operable and inoperable non-small cell lung cancer. J Cancer Res Clin Oncol.

[11] Zhang F, Sun P, Wang ZQ, Wang DS, Wang Y, Zhang DS, Wang FH, Fu JH, Xu RH, Li YH (2016). Low preoperative albumin-globulin score predicts favorable survival in esophageal squamous cell carcinoma. Oncotarget.

[12] Arigami T, Okumura H, Matsumoto M, Uchikado Y, Uenosono Y, Kita Y, Owaki T, Mori S, Kurahara H, Kijima Y, Ishigami S, Natsugoe S (2015). Analysis of the fibrinogen and neutrophil-lymphocyte ratio in esophageal squamous cell carcinoma. Medicine (Baltimore).

[13] Fearon KC, Falconer JS, Slater C, McMillan DC, Ross JA, Preston T (1998). Albumin synthesis rates are not decreased in hypoalbuminemic cachectic cancer patients with an ongoing acute-phase protein response. Ann Surg.

[14] McMillan DC, Watson WS, O'Gorman P, Preston T, Scott HR, McArdle CS (2001). Albumin concentrations are primarily determined by the body cell mass and the systemic inflammatory response in cancer patients with weight loss. Nutr Cancer.

[15] Matsuda S, Takeuchi H, Kawakubo H, Fukuda K, Nakamur R, Takahashi T, Wada N, Saikawa Y, Omori T, Kitagawa Y (2015). Cumulative Prognostic Scores Based on Plasma Fibrinogen and Serum Albumin Levels in Esophageal Cancer Patients Treated with Transthoracic Esophagectomy: Comparison with the Glasgow Prognostic Score. Ann Surg Oncol.

[16] Takeno S, Hashimoto T, Shibata R, Maki K, Shiwaku H, Yamana I, Yamashita R, Yamashita Y (2014). The high-sensitivity modified Glasgow prognostic score is superior to the modified Glasgow prognostic score as a prognostic predictor in patients with resectable gastric cancer. Oncology.

